# Pain Assessment–Can it be Done with a Computerised System? A Systematic Review and Meta-Analysis

**DOI:** 10.3390/ijerph13040415

**Published:** 2016-04-13

**Authors:** Nuno Pombo, Nuno Garcia, Kouamana Bousson, Susanna Spinsante, Ivan Chorbev

**Affiliations:** 1 Instituto de Telecomunicações (Telecommunications Institute), University of Beira Interior, Covilhã 6200-001, Portugal; ngarcia@di.ubi.pt; 2 Department of Informatics, University of Beira Interior, Covilhã 6200-001, Portugal; 3 ALLab—Assisted Living Computing and Telecommunications Laboratory, University of Beira Interior, Covilhã 6200-001, Portugal; 4 Department of Aerospace Sciences, University of Beira Interior, Covilhã 6200-001, Portugal; bousson@ubi.pt; 5 Dipartimento di Ingegneria dell’Informazione, Università Politecnica delle Marche, Ancona 60121, Italy; s.spinsante@univpm.it; 6 Faculty of Computer Science and Engineering, Ss. Cyril and Methodius University Skopje, Skopje 1000, Macedonia; ivan.chorbev@finki.ukim.mk

**Keywords:** mhealth, pain diaries, pain scales, pain assessment, chronic pain

## Abstract

*Background*: Mobile and web technologies are becoming increasingly used to support the treatment of chronic pain conditions. However, the subjectivity of pain perception makes its management and evaluation very difficult. Pain treatment requires a multi-dimensional approach (e.g., sensory, affective, cognitive) whence the evidence of technology effects across dimensions is lacking. This study aims to describe computerised monitoring systems and to suggest a methodology, based on statistical analysis, to evaluate their effects on pain assessment. *Methods*: We conducted a review of the English-language literature about computerised systems related to chronic pain complaints that included data collected via mobile devices or Internet, published since 2000 in three relevant bibliographical databases such as BioMed Central, PubMed Central and ScienceDirect. The extracted data include: objective and duration of the study, age and condition of the participants, and type of collected information (e.g., questionnaires, scales). *Results*: Sixty-two studies were included, encompassing 13,338 participants. A total of 50 (81%) studies related to mobile systems, and 12 (19%) related to web-based systems. Technology and pen-and-paper approaches presented equivalent outcomes related with pain intensity. *Conclusions*: The adoption of technology was revealed as accurate and feasible as pen-and-paper methods. The proposed assessment model based on data fusion combined with a qualitative assessment method was revealed to be suitable. Data integration raises several concerns and challenges to the design, development and application of monitoring systems applied to pain.

## 1. Introduction

Chronic pain accounts for billions of dollars in annual medical expenditures [1]; in addition to that, the resulting decreased workers’ productivity contributes to indirect costs [2,3,4], and the loss of quality of life has to be mentioned as a critical related effect. As chronic pain persists over a long period of time [5], its management results expensive due to the need of long-term rehabilitation and multi-disciplinary treatments [6]. In fact, the chronic condition of pain is determined by an arbitrary interval which may vary between twelve weeks, and six months [5]. However, it’s hard to come up with an immediate and precise assessment that leads to the right treatments, avoiding inadequately assessed and undertreated cases [7,8]. Firstly, pain is a highly subjective experience for each individual [9]. Secondly, due to its duration, the assessment is often accomplished at the patient’s home, and this represents a challenge for the accuracy of the treatment and the cost-effectiveness of the monitoring. Therefore, as self-report is considered the most accurate pain assessment method [10,11], patients should be asked to periodically rate their pain severity and related symptoms. Unsurprisingly, in the last few years, handheld devices and Internet-delivery treatment (IdT) methods were increasingly used to enable chronic pain monitoring. These systems were used for many different purposes [12], including education, reminders, feedback (in both directions between healthcare professionals and patients), and disease control. 

The ubiquity of mobile devices and the Internet raised the paradigm of the new care model based increasingly more on contacts rather than visits [13]. In fact, the ability to interact with the system anywhere and anytime thoroughly changes the coordinates of time and place, and offers invaluable opportunities for new approached to healthcare delivery. Moreover, mobile devices have shown significant advances in storage capacity, battery efficiency, portability [14] and the ability to access internet-based resources [15], therefore increasing their suitability for use in healthcare systems. The adoption of technology has allowed the development of electronic pain diaries (ED) as a computerised version of paper pain diaries (PD). These systems enable patients either to report complaints close in time to the event causingpain, called ecological momentary assessment (EMA), or to address retrospective pain, that consists in pain recall over some period of time. Rather than an isolated value, pain results from multiple aspects [16,17,18,19,20], such as sensory (e.g., location, intensity), affective (e.g., depression, anxiety) and cognitive (e.g., quality of life) ones. For this reason, chronic pain patients are invited to answer many questionnaires and scores (e.g., McGill Pain Questionnaire, Visual Analogue Scale), and/or to adopt specific behaviours as a way to treat their pain in all its dimensions. For example, the monitoring program may include self-monitoring of pain, adherence to prescribed medications, regular exercise, and weight control. In summary then, the monitoring of chronic pain patients leads to many challenges across a range of topics such as technology (e.g., to collect and send data), clinical settings (e.g., duration of treatment, momentary pain or recall pain), and multi-dimensional pain assessment (e.g., questionnaires, scales).

The aims of this study are to describe ED implemented through mobile and web-based systems applied to chronic pain monitoring, and to determine the benefits obtained from adopting these technologies, in comparison to traditional pen-and-paper methods. This is carried out by means of an extensive review of the English-language literature about computerised systems related to chronic pain complaints.

## 2. Materials and Methods

### 2.1. Research Questions

The primary question in this review was (RQ1) Can ED replace PD for patients’ monitoring? The secondary questions were (RQ2) which ubiquitous systems have been used in the monitoring of chronic pain patients? and (RQ3) which data (e.g., questionnaires and scales) are collected?

### 2.2. Inclusion and Exclusion Criteria

Studies were included in this review when they met the following criteria: (1) they dealt with computerised systems related to chronic pain complaints, (2) they included data about pain assessment, namely pain intensity; and (3) they were conducted using electronic means that included mobile devices (e.g., smartphone, Personnal Digital Assistant (PDA), tablet Personnal Computer) or web-based forms; (4) preliminary or definitive results were presented; and (5) they were written in English. These criteria were also applied to studies obtained from reference tracking. Reviews, study protocols, and researches where data acquisition relied exclusively on e-mails or chats were excluded. There were no age or disease restrictions: participants could be either adults or children, they might comprise chronic pain patients or healthy individuals with pain complaints. 

### 2.3. Search Strategy

The team conducted a systematic search over the following electronic databases: BioMed Central, Pubmed Central, and ScienceDirect. Only the studies published from 2000 up until 30 June 2012 meeting the inclusion criteria were included. Every study was independently evaluated by two reviewers (Nuno Pombo and Nuno Garcia) and its suitability determined with the agreement of both parties. A third reviewer was considered to adjudicate on differences of opinion, but it was not required because a consensus was reached. The studies were also examined to identify and isolate clusters reporting the same data, so as to avoid the risk of bias [21]. 

### 2.4. Extraction of Study Characteristics

The data extracted from the studies, were tabulated (see Appendix A1) and grouped into mobile and web-based systems. For each study, details about year of publication, age of studied population (median and standard deviation (SD)), and number of participants were reported. The data managed (collected and/or complementary) by the system were grouped into three categories: pre-treatment (data obtained during the recruitment of participants were excluded), treatment and post-treatment (also includes follow up). However, data related to intervention quality and satisfaction assessment were omitted from this review. Finally, the meta-analysis included studies comprising randomised controlled trials (RCTs) that evaluated the usage of ED or IdTs and presented pre- and post-treatment comparisons. A mathematical model was used (see Section 2.7.1) to determine the effect of technology in the monitoring of pain. Firstly, the pain outcomes obtained in the RCTs’ groups (intervention and control) were converted to a 0–100 scale. Secondly, a qualitative assessment (see Section 2.7.2) was performed to build an oriented analysis on pain intensity. 

### 2.5. Quality Assessment

The methodological quality of all the studies was independently assessed by the two reviewers using a list of 10 criteria, which was formulated for the purpose of this study (see Appendix A2). Each criterion was rated as either poor/absent (=0), reasonable (=1), or good (=2). Items scores were summed to obtain a total study quality score (range 0–20). As shown in Table A1, the quality sum scores were used to classify studies into two groups, above or below an average quality threshold. 

### 2.6. Risk of Bias Assessment

Two reviewers (Nuno Pombo and Kouamana Bousson) independently assessed the risk of bias of each RCT included in the meta-analysis (see Table A2), using the Cochrane Collaboration’s risk of bias tool [22]. Distinct domains were evaluated, such as: the method used to generate and to conceal the allocation sequence, the blinding of participants, personnel and outcome assessors, incomplete outcome data, selective outcome reporting and other sources of bias. 

### 2.7. Mathematical Analysis

#### 2.7.1. Statistical Data Fusion

The mathematical model is based on the data fusion methods described in [23,24,25] and summarized below. 

Let us consider *n* sets of data samples, each of which has a Gaussian distribution N (${\bar{x}}_{i}$, σ*_i_*), where ${\bar{x}}_{i}$ and σ*_i_* are respectively the mean (or mathematical expectation) and the standard deviation of samples in the set *i*. Then, the probability distribution of the aggregated set is again Gaussian with a mean ${\bar{x}}_{i}$ and a standard deviation σ computed as:

(1) $$\bar{x}=\overset{n}{\underset{i=1}{\sum }}a_{i}x_{i}=\alpha \overset{n}{\underset{i=1}{\sum }}\frac{x_{i}}{\sigma_{i}^{2}}$$

where *a_i_* is defined by:

(2) $$a_{i}=\frac{1}{\sigma_{i}^{2}}\alpha ,\;i=1,\ldots ,n$$

(3) $$\text{and }\alpha ={\left(\frac{1}{\sigma_{1}^{2}}+\frac{1}{\sigma_{2}^{2}}+\ldots +\frac{1}{\sigma_{N}^{2}}\right)}^{-1}$$

(4) $$\text{and }\sigma^{2}=\overset{N}{\underset{i=1}{\sum }}a_{i}^{2}\sigma_{i}^{2}$$

#### 2.7.2. Qualitative Analysis

**Figure 1:**
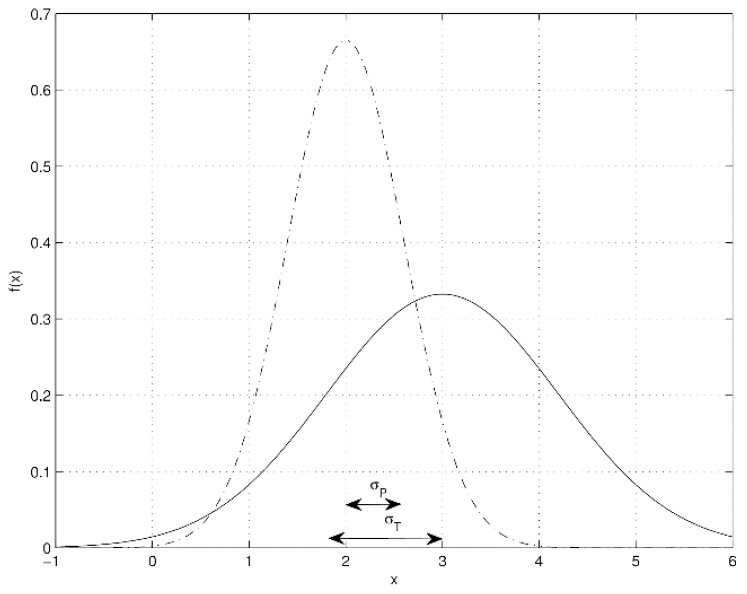
Example of a distribution curve when technology and pen-and-paper are qualitatively equivalent.

The mean and the standard deviation, computed as described in the last section, are used for the qualitative analysis method, that we proposed below, which aims to produce a more accurate outcome. Let us consider:

σ*_T_*: standard deviation of technology outcome;

σ*_P_*: standard deviation of pen-and-paper outcome;

${\bar{x}}_{T}$: mathematical expectation of technology outcome;

${\bar{x}}_{P}$: mathematical expectation of pen-and-paper outcome;

Consider furthermore the following conditions:

Condition (P): ${\bar{x}}_{P}\;\in \left({\bar{x}}_{T}-\sigma_{T},{\bar{x}}_{T}+\sigma_{T}\right)\;or\;{\bar{x}}_{T}\;\in \left({\bar{x}}_{P}-\sigma_{P},{\bar{x}}_{P}+\sigma_{P}\right)$ for instance as shown in Figure 1 where ${\bar{x}}_{T}=3,{\bar{x}}_{P}=2,\sigma_{T}=1.2,\sigma_{P}=0.6$

**Figure 2:**
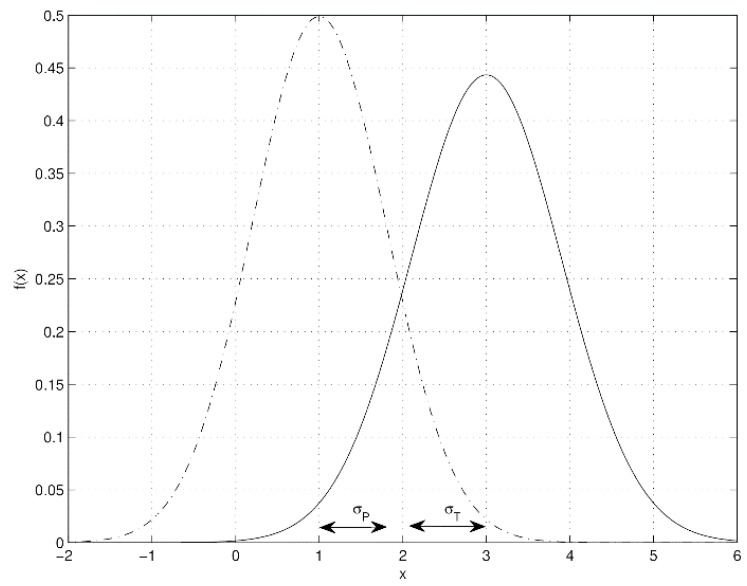
Example of a distribution curve when technology and pen-and-paper are qualitatively different.

The opposite condition is presented in Figure 2 with${\bar{x}}_{T}=3,{\bar{x}}_{P}=1,\sigma_{T}=0.9,\sigma_{P}=0.8$. The rationale of condition (P) is that since the standard deviation σ is the average magnitude of the sample dispersion with respect to its mean value $\bar{x}$ (mathematical expectation), any value *x* that is located at a distance from $\bar{x}$ less than the standard deviation (that is, |*x* − $\bar{x}$| < σ) may be considered as qualitatively equal to $\bar{x}$. Using condition (P) described above, a qualitative analysis is performed to clarify which one among technology and pen-and-paper approach provides the best way to get fair results in pain monitoring.

*Case 1*: when the lower mean value (mathematical expectation) implies better results:

If condition (P) is verified, then using technology or pen-and-paper gives rise to the same conclusion, even though the mean values may be different; else if (${\bar{x}}_{T}$ < ${\bar{x}}_{P}$) then technology provides better results than pen-and-paper; else pen-and-paper provides better results than technology.

*Case 2*: when the higher mean value (mathematical expectation) implies better results:

If condition (P) is verified, then using technology or pen-and-paper gives rise to the same conclusion, even though the mean values may be different; else if (${\bar{x}}_{T}$ > ${\bar{x}}_{P}$) then technology provides better results than pen-and-paper; else pen-and-paper provides better results than technology.

#### 2.7.3. Considerations for the Analysis

Several studies were excluded from this analysis due to the absence of comparisons between pre-treatment and post-treatment outcomes [26,27,28,29,30,31,32,33], or the absence of technology validation purposes [34]. The remaining sixteen unique studies were assessed in terms of risk of bias (see Appendix A3). Three studies [35,36,37] were appraised to be at lowest risk of bias, as they met every criterion except the blinding of participants, personnel and outcome assessors. In fact, none of the included RCTs met this criterion. The lack of information and explanation for attrition and missing data was observed, whereas all studies clearly reported the different outcomes. These outcomes were used to implement statistical analysis across the included RCTs. During the analysis, one study was excluded due to the inexistence of SD in the reported data [38]. In addition, several studies were partially excluded due to high SD in some outcomes (a.k.a. outliers) [39,40], or due to unfeasible conversion from t-scores to a continuous scale [35]. Instead of a single analysis of the studies, the pre- and post-treatment data obtained from intervention groups and control groups across the different RCTs were combined using data fusion methods [23,24,25]**,** and compared to produce a more accurate conclusion. Thus, as shown in Table 1, the adoption of technology related with pain intensity was assessed. 

## 3. Results

**Figure 3:**
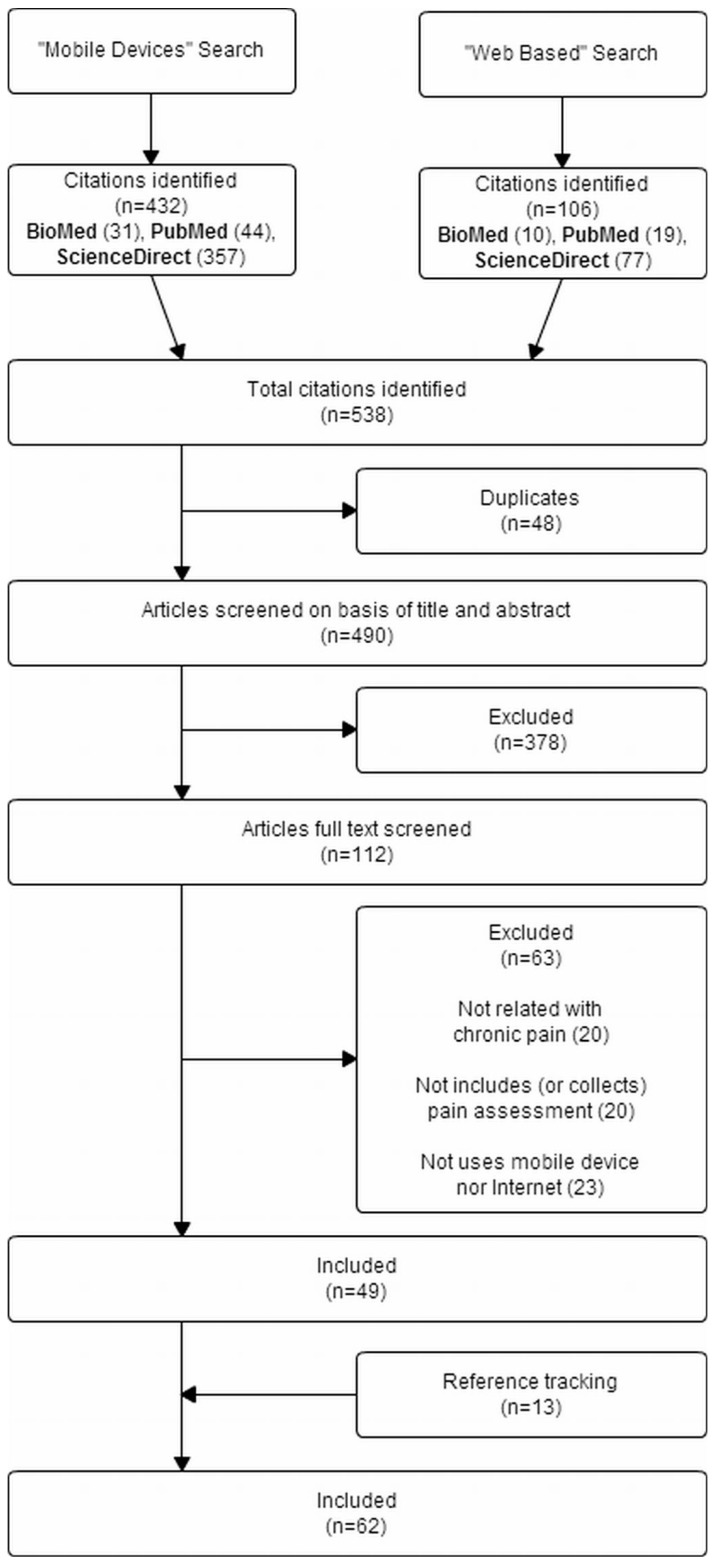
Selected studies.

As illustrated in Figure 3, 490 unique citations were identified, of which 378 were excluded as a result of screening, in terms of title, abstract, and keywords. The remaining 112 papers were full text evaluated, which resulted in the exclusion of 63 papers that did not match the defined criteria. Furthermore, the reference tracking allowed for the inclusion of 13 additional papers. In summary then, our review examined 62 papers, representing 55 unique studies, due to the fact that studies reporting the same data were clustered to avoid risk of bias.

The included studies encompass a total of 13,338 participants distributed by 43 studies (78%) related to mobile systems, and 12 (22%) studies dealing with web-based systems. Eighty-one percent of mobile systems (35 studies) were designed to enable usage in patients’ home whereas the remaining eight studies limited their use within hospital facilities. The data were collected at intervals or during the clinical visit or at the end of the study, and transmitted to the system database by different channels, such as: Internet, SMS, or cable. Web-based systems were reported in 12 studies varying between online questionnaires and cognitive-behavioural therapy (CBT). Moreover, 10 studies (83%) used phone calls, SMS or emails as a complement of the IdT. This methodology aims to remind patients to collect data, support system handling, and to establish contact between healthcare professionals and patients.

Moreover, 16 out of the 55 studies (29%) included in this review were published before or during 2006, and among the remaining 39 studies, 27 studies were published between the beginning of 2008 and the end of 2010. Thirty-two studies (58%) included complementary data, obtained outside the system in at least one of the following phases: pre-treatment (28 studies), treatment (8 studies) or post-treatment (16 studies).

The most representative objective was validating the IdT (12 studies, 22%), the assessment of ED (12 studies), the comparison between ED and PD (nine studies), the comparison between recalled pain and EMA (six studies), and the evaluation of medication in treatment of patients suffering from pain (three studies). Eight studies reported the correlation of several pain conditions, namely: physical activity, relationship, emotional distress, fear, and sleep.

The CBT was presented in 19 studies, among which seven were related to mobile systems. The remaining 12 studies presented CBT as a support of IdT, and included tailored exercises according to participants’ symptoms, multimedia content, information and lessons about physical, cognitive, behavioural and motivational topics. The main principles of CBT for chronic pain management are based on helping the patient understand how much pain is experienced, coping-skills training, and cognitive restructuring affected by cognition and behaviour [49].

### 3.1. Mobile Systems

Forty-three studies were related to mobile systems, out of which 35 (81%) were designed to allow their usage in patients homes during at least one phase of the intervention (pre/post-treatment, treatment). The remaining eight studies were limited to the use of the proposed system in hospital facilities during patients’ visits and thereby only comparisons among sporadic records collected during the treatment period were provided. Meanwhile, 19 studies presented transmission of data to a remote server immediately after its edition. Three studies did not report this process, whereas 21 studies reported elapsed time between the editing and the subsequent delivery. Thus, data were collected at intervals, or during the clinic visit, or at the end of the study. Internet was the preferred channel for sending data (14 studies), followed by uploading through personal computer (nine studies), and SMS (three studies). Data transmission after its edition may allow real-time access to physicians, and therefore, clinical decisions supported by updated information on the patient’s conditions. Moreover, undelayed data transmission may provide the enforcement of triggering messages and alerts according to the reported pain values. This method was highlighted by four studies and comprised a clinical session report generation, SMS alerts according to answers and warning messages about the activity patterns, displayed in PDA. Data storage in a Personal Health Record (PHR), wrist actigraphy in sleep assessment, and activity monitoring supported by a Body Area Network (BAN) were proposed in a single study respectively. Interactive voice recorder (IVR) was referred in two studies [44,50]. Time of intervention ranged from one clinical session to 52 weeks (one year).

### 3.2. Web-Based Systems

Web-based systems were reported in 12 studies, out of which 11 consisted in RCTs, comprising two groups of participants called: intervention group (IG) and control group (CG). The difference between them is that a web site was used to deliver the treatment to IG participants. At the end of intervention, participants of both groups were assessed and the IdT effects were determined. The IdT consisted of online questionnaires and/or CBT. All the articles reported positive effects and improvement in health status. With the exception of [37,39], all web-based systems used emails or phone calls jointly with Internet (83%). Six studies adopted e-mails [41,42,45,46,48,51] and three of them also performed phone calls [45,46,48], to remind patients to use and/or interact with the system. In addition, emails were applied to obtain data [40,45,46,51], to support the system handling [36,41], and, together with phone calls, to establish a contact between healthcare professionals and patients [36,43]. One study [40], allowed phone calls to support the system handling. Finally, [52] used SMS to remind patients to collect data. In the same study, mobile phones with Internet access were used to present a web site whereupon treatment was provided, and therefore, it has been classified as a web-based system. Time of intervention ranged from 3 to 52 weeks (one year). It should be noted that remote data transmission is not required in these systems, while it occurs in mobile monitoring applications. 

### 3.3. Meta-Analysis

The qualitative and quantitative analysis (see Section 2.7) revealed that the benefits of technology and pen-and-paper are equivalent on pain intensity (48.67 ∈ (50.98 ± 3.35) and 50.98 ∈ (48.67 ± 3.49)), as presented in Table 1. Firstly, the proposed statistical data fusion model processes each study as a different sensor and computes the individual mean and SD, related with the processed variable (determined by the collected data from questionnaires and/or scores) in both arms of the study (pen-and-paper group and computerised system group). Secondly, the combination of these values resulted in an aggregate value. Thirdly, the fusion model computes all aggregate values and presents a final decision according to the rules defined in Section 2.7.

## 4. Discussion

Some potentials and risks related to mobile and web-based systems were evidenced from the full text evaluation of included studies. Firstly, the usage of ED produces more reliable data compared to PD. Secondly, ED and IdT result in real-time analyses and subsequent agile treatment adjustments. Thirdly, ED and IdT provide time-saving and enable cost-effective medical practices. Nevertheless, training for clinical staff is critical [53], and strongly recommended to promote standardised procedures and adherence [54]. In addition, device failures considered in system design [55], should be addressed to avoid missing values and/or prolonged data editing. It should be noticed that due to the frequent loss of mobile devices, their use to store health records implies the risk of losing data and personal information. These topics, along with the inefficient use of collected data to improve treatment effectiveness, emerged as critical limitations. This review included 19 studies related to CBT, in which the following outcomes were observed: the effectiveness for decreasing chronic pain, in line with [49,56,57], the reduction of pain related behaviours as suggested by [58,59], and a facilitated return to work, as presented by [60,61]. In spite of their absence in these studies, innovative CBT, such as: serious games [62,63] and augmented reality [64,65], seem to be promising. Serious games are the application of motivational aspects of gaming to encourage positive health behaviours [66], whereas augmented reality provides virtual environments combined with touch sensations resulting from interacting with real objects [67]. Further work is needed to understand how these technologies can aid the transformation of CBT delivery models.

The use of SMS [68] to collect data, as proposed by [69,70,71,72], and to deliver CBT, as suggested by [52], may improve treatment outcomes, due to the fact that tailoring messages to individuals may lead to effective health behaviour changes [73,74,75]. Only one study [76], mentioned data integration with other systems such as PHR, which suggests limitations on accessing the collected data. In addition, some mobile-based systems were designed to interact directly with patients without the presence of a healthcare professional [77,78] and/or without evidence of reliability and accuracy. However, as pain is a multifaceted experience, its therapy tends to involve many healthcare professionals and different expertises whereby the data integration may result in the reduction of not regulated self-diagnosis [79]. Therefore, it is desirable that patient information may be obtained and delivered both easily and safely (e.g., avoidance of medical examination redundancy, faster patient profile acquisition, and permanent storage of clinical records) which raises some concerns and challenges related to security aspects such as privacy and confidentiality [80], and reliable communication methods between healthcare professionals and patients. 

In line with this, cloud computing as an emerging technology that provides elastic infrastructure, and efficient resource utilization [81], appears to be a promising solution for design, development and integration of systems. This technology may enable scalable, portable, and interoperable mobile and web-based systems as to deliver clinical solutions to the patients, anytime and anywhere [82]. In addition, social media websites have been useful in the last few years to improve networking and communication [83] (e.g., Facebook, Twitter), and represent a new source of information and knowledge. Therefore, it is expected that clinical systems will advance to interact with patients via social media, so as to provide CBT, serious games, self-help, symptoms information and multimedia content. Thus, new studies should be addressed to determine the real benefits and disadvantages of treatments delivery using social media. Furthermore, complementary studies should be carefully addressed to analyse both data and patient privacy. 

Finally, our meta-analysis demonstrated that the effects of technology and pen-and-paper should be obtained not only based on the comparison of the standard deviations together with the values of the mathematical expectations, but also considering the condition (P) described in Section 2.7. In addition, the outcome of our meta-analysis is highly accurate as evidenced by the lower SD of the obtained aggregated values (resulting from the computation of the SD of all the included samples) compared with the individual SD presented in each study. In fact, we found that technology and pen-and-paper present equivalent outcomes suggesting not only that technological systems are feasible, but also there is room for improvement to produce significant effects in patients’ conditions and welfare. Moreover, further studies should be promoted to determine not only the effect of technology on different dimensions of pain (e.g., anxiety, depression, catastrophizing, disability and interference) but also the side effects of the application of technology in economic, medical, educational, and social domains.

## 5. Conclusions

This review distinguished mobile and web-based systems related to chronic pain complaints. Sixty-two studies were examined and the main findings are summarised as follows:

(RQ1) The qualitative analysis model, stemming from the data fusion method, combined with a quantitative model, based on the comparison of the standard deviations together with the values of the mathematical expectations, revealed that technology is equivalent to pen-and-paper in terms of effect on pain intensity monitoring;

(RQ2) Sixty-two studies were included encompassing 13,338 participants. A total of 50 (81%) studies related to mobile systems, and 12 (19%) related to web-based systems;

(RQ3) The data extracted from the included studies, revealed the use of almost ninety different scales and questionnaires at pre/post/during treatment. The data collected comprised, among others: location, duration, and intensity of pain, consequences as the impact on quality of life, emotional and aversive aspects. This highlights the multi-dimensional nature of pain.

Despite these findings, effects of technology on practitioners and patients outcomes remain understudied, and their promise to increase self-care and accurate monitoring remains mostly untested. In addition, data integration raises several concerns and challenges to the design, development and application of monitoring systems applied to pain.

### Limitations

Some limitations of this review should be mentioned. First, only English-language publications were included. Second, the lack of technical explanations related to data acquisition, transmission and storage, restricted both analysis and extraction. Third, the null hypothesis was considered, that means, all sample data are assumed to be sufficient.

## Figures and Tables

**Table 1:** Comparison between pen-and-paper, and mobile and web technology using pre and post treatment results by study and overall.

Pain intensity										
Study	Variable	Technology		Pen and Paper		Technology		Pen and Paper		Favourable To
		Pre Treatment	Post Treatment	Pre Treatment	Post Treatment	Aggregated Value	SD	Aggregated Value	SD	
		Value (SD)	Value (SD)	Value (SD)	Value (SD)					
Berman [41]	BPI (mean)	52 (19.40)	45.60 (18.30)	54.30 (17.40)	47.30 (18.40)	48.61	13.31	51	12.64	Technology
Buhrman [36]	Pain (mean)	37.40 (18.20)	34.30 (16.80)	44.4 (14.20)	39.6 (16.30)	35.73	12.34	42.33	10.71	Technology
	MP—pa in severity	63.33 (31.67)	40 (18.33)	83.33 (28.33)	53.33 (13.33)	45.86	15.87	58.77	12.06	Technology
Devineni [42]	Headache pain	31.80 (17)	18.60 (13)	35.50 (15.50)	30.60 (14.70)	23.47	10.33	32.92	10.67	Technology
Hicks [43]	Pain (mean)	48 (13)	34 (24)	43 (16)	47 (22)	44.82	11.43	44.38	12.94	Pen-and-Paper
Litt [44]	MPI (mean)	43.83 (21)	20.50 (16.33)	35.17 (14.33)	25 (22.67)	29.29	12.89	32.26	12.11	Technology
Ljótsson [45]	Pain	65 (42.50)	35 (37.50)	60 (37.50)	60 (40)	48.13	28.12	60	27.36	Technology
Lorig [39]	Pain	65.30 (22.70)	58.60 (24.40)	63.70 (22.20)	63.40 (23.10)	62.19	16.62	63.56	16.01	Technology
Palermo [37]	Pain	54.50 (22.50)	35.40 (24.20)	51.70 (16.50)	47.60 (18.40)	45.64	16.48	49.87	12.28	Technology
	Retrospective pain	66.30 (18.70)	49.60 (21.80)	61.60 (18.40)	54.50 (20.40)	59.22	14.19	58.42	13.66	Pen-and-Paper
Ruehlman [46]	PCP-S—pain severity	76.47 (9.72)	71.10 (12.94)	74.78 (10.91)	71.66 (13.28)	74.53	7.77	73.52	8.43	Pen-and-Paper
Turner [47]	Pain (mean)	43 (22)	39 (24)	43 (19)	40 (22)	41.17	16.22	41.72	14.38	Technology
Williams [48]	BPI—pain severity	51 (14)	43 (16)	49 (14)	49 (15)	47.53	10.54	49	10.23	Technology
**Fusion**	value	**55.90 (4.80)**	**40.57 (5.08)**	**52.65 (4.56)**	**49.02 (4.94)**	**48.67**	**3.49**	**50.98**	**3.35**	**Equivalent**
	alpha	23.04	25.82	20.79	24.38					

## Abbreviations

The following abbreviations are used in this manuscript:

CBT: Cognitive-behavioural Therapy
CG: Control Group
ED: Electronic pain diaries
EMA: Ecological Momentary Assessment
IdT: Internet-delivery treatment
IG: Intervention Group
PD: Paper pain diaries
PHR: Personal Health Record
RCT: Randomised controlled trial
SD: Standard Deviation

## Appendix A1

**Table A1:** Studies characteristics.

Study/Year	Population Participants (Mean Age, SD)	Patient Home	Data			Quality
			As a Complement to the System	Collected through the Use of System	Transmission	
**Mobile systems**						
Allen [84,85], 2009	157 (61.7 ± 10.6)	Yes	**Pre:** CSQ	Pain intensity (VAS), immediately after waking, then approximately every 2 h throughout the day (in order to complete at least 7 pain ratings per day) and immediately before going to sleep (to recall the average pain during the day)	NR	L
Anatchkova [86], 2009	100	No		Pain intensity (NRS), computer adaptive dynamic assessment of The Chronic Pain Impact Item Bank [87], and SF-12, in the medical appointment	NR	L
Axen [69,70], 2011	262 (44)	Yes	**Pre:** Pain intensity (NRS), location, duration and frequency, self-rated general health (5-point Likert scale). EuroQoL 5 (EQ5D) **Post:** EQ5D and self-rated general health (6-months follow up)	Pain intensity (NRS), once a week using SMS	Instant	L
Badr [88], 2010	54 patients (49.4 ± 10.8) 48 partners (51.3 ± 11.5)	Yes		**Patients:** pain intensity (NRS), mood, medication taken and pain relief, 6 times per day between 9 a.m. and 9 p.m. Perceptions of relationship functioning in the last assessment of the day. **Partners:** patients’ pain, own mood and perceptions of relationship functioning, at similar time points	Instant	L
Baron-Mahn [89,90], 2009	2094 painful radiculopathy (59.4 ± 14.4) 1623 painful diabetic neuropathy (61.9 ± 13.0) 498 postherpetic neuralgia (60.6 ± 15.4)	No		MOS-SS, PHQ, PD-Q and pain location (pinpointed in 3D mannequin) in the medical appointment	Delayed	L
Broderick-Schneider [50,91,92], 2008	83 (56.2 ± 11.1)	Yes	**Treatment:** 10 random recalls pain assessment via phone interview (interactive voice recording was used) **Post:** Pain Intensity (VAS)	SF-36, BPI, BFI, MPQ, 7 times per day during the patients’ waking hours	Delayed	L
Clauw [34], 2008	399 IG 100 mg/day (49.5 ± 10.9) 396 IG 200 mg/day (50.4 ± 10.6) 401 CG (50.7 ± 10.4)	Yes	**Pre:** FIQ, MASQ, MOS-SS, MDHAQ, MFI, BDI, and ASEX **Treatment:** 3, 7, 11 and 15 week visit: PGIC, SF-36, FIQ, MASQ, MOS-SS, MDHAQ, MFI. BDI and ASEX only at week 15	**Diary:** pain intensity (VAS), 5 times per day (morning, 3 during day and evening) **Weekly:** pain, fatigue, influence of pain in self-care (VAS)	Instant	H
Connelly [93], 2010	9 (12.3 ± 3.4)	Yes		**Children:** pain intensity (VAS), PANAS-C, CALQ, 3 times per day (morning, afternoon, and evening) **Parents:** PANAS, ARCS at the same time points, using a separate PDA	Delayed	L
Gaertner [94], 2004	24 (49.9 ± 15.1) Crossover randomized between IG and CG	Yes		Pain intensity (NRS), once a day and symptom assessment (fatigue, nausea, dyspnea, weakness…), once a week	Delayed	L
Ghinea [95], 2008	45 (46.1)	Yes		Pain intensity (VAS) and location (pinpointed in 3D mannequin), 3 times a day	Instant	L
Giske [72], 2010	50 (50.0 ± 11.0)	Yes	**Pre:** HSCL-25, FIQ **Post:** Pain intensity (VAS) and pain location	Pain intensity (NRS), 5 times a day between 9 a.m. and 9 p.m., using SMS	Instant	L
Heiberg [96], 2007	38 (58.4 ± 12.9)	Yes		**Diary:** pain intensity (VAS), fatigue, and patient global evaluation of their disease, RADAI, 4 times per day **Weekly:** MHAQ, SF-36	Instant	H
Jamison [28], 2001	20 IG (42.1 ± 5.0) 16 CG (43.3 ± 9.2)	Yes	**Pre:** CPEQ, SCL-90 **Treatment:** MPQ-SF (once a month). Pain reported weekly by phone interview **Post:** SCL-90	Pain intensity (VAS) and pain ratings of the previous 16 waking hours, once a day (bedtime)	Delayed	H
Jamison [97], 2002	24 (34.4)	No		Pain intensity (VAS)	Delayed	L
Jamison [98], 2006	21 (42.0 ± 4.9)	Yes	**Pre:** CPEQ, SF-36, MPQ-SF, SCL-90 **Treatment:** Pain reported weekly by phone interview	Pain intensity (VAS), at least once a day	Delayed	H
Jamison-Wasan [26,27], 2010	21 IG ED + CBT (47.0 ± 7.8) 21 CG #1 ED (46.6 ± 6.8) 20 CG #2 ED (49.6 ± 6.8)	Yes	**Pre and Post:** ABC, BPI, COMM, HADS, MINI, PDI, SOAPP-R **Post:** PDUQ	BPI, pain location once a month at clinic visit Wasan’s study, also includes four questions to assess craving for prescription opioids over the past 24 h (14 days ED at patients' home) **CBT:** Group educational sessions (e.g., opioid addiction risks and medication compliance, making lifestyle changes...) and individual motivational counseling (review of medication adherence, support for patients’ efforts, education on pain management and drug misuse...)	Delayed	H
Jespersen [71], 2012	188 (44.4 ± 9.0)	Yes	**Pre:** AMS	AMS, IPAQ, once a week using SMS	Instant	H
Koroschetz [99], 2011	1623 painful diabetic neuropathy (61.9 ± 13.0) 1434 fibromyalgia (51.9 ± 10.8)	No		MOS-SS, PHQ, PD-Q and pain location (pinpointed in 3D mannequin) in the medical appointment	Delayed	L
Kvien [100], 2005	30 (61.6)	No		Pain intensity (VAS), fatigue, and patient global evaluation of their disease, RADAI, MHAQ, SF-36, at 2 medical appointments	Instant	L
Lewandowski [101], 2010	39 chronic pain (15.3 ± 1.5) 58 healthy participants (14.7 ± 1.8)	Yes	**Pre:** CES-D	Sleep quality (NRS) in the morning and pain intensity (NRS) in the evening. Integrated with wrist actigraphy to monitorize the sleep	Delayed	L
Levin [102], 2006	24	Yes		Pain intensity (NRS), location, duration reported via automated speech telephony delivery (a.k.a automated speech recognition)	Instant	L
Li [103], 2010	60 (69.0 ± 10.0)	Yes	**Pre and Post:** MPQ-SF	MPQ-SF, 8 times per day (hourly between 2 and 9 p.m.)	Delayed	H
Lind [104], 2008	12 (67.5 ± 7.8)	Yes		Pain intensity (VAS), 3 times a day (8 a.m., 1 p.m., 8 p.m.)	Instant	L
Litt [44], 2009	32 IG 22 CG Overall (41.0 ± 11.9)	Yes	**Pre and Post:** MPI, CES-D	Pan location, unpleasantness experienced, perceived control over pain, catastrophization and coping, 4 times per day (from 8 a.m. to 10 p.m.). Interactive voice recording was used **CBT:** relaxation training, cognitive restructuring and stress management	Instant	H
Luckmann [76], 2010	4	Yes		Pain intensity (NRS), location, activity and treatment completed each 2–4 waking hours. Acute pain registered when happens. Sleep report in the morning and end of day report before sleep. Data integration with PHR	Instant	L
Marceau [105], 2010	67 IG (48.5 ± 11.6) 67 CG (50.5 ± 11.0)	No		BPI at each monthly clinic visit. Pre and post-treatment and 5-month follow up: BPI, PCS, ODI, CES-D	Instant	H
McClellan [29], 2009	9 IG 10 CG Overall (13.4 ± 2.9)	Yes		Pain intensity at morning and evening (10-point Likert scale), pain location, sleep quality, and functional limitations once a day **CBT:** coping skills program, once a day. Parents presence is allowed	Instant	H
Oerlemans [38], 2011	37 IG (35.9 ± 11.7) 39 CG (40.6 ± 15.5)	Yes	**Pre and Post (upon treatment and 3-month follow up):** Pain intensity (5-point Likert scale), CFSBD, IBS-QoL, PCS	Pain intensity (5-point Likert scale) 3 times per day (morning, afternoon and evening). Sleep quality and intended activities for the day. (morning), accomplished activities, cognitions, and feelings (afternoon), and satisfaction with activity level and achievements of that day (evening) **CBT:** situational feedback on their diaries from a psychologist	Instant	H
Okifuji [106], 2011	81 (28.8 ± 6.2)	Yes		Overall pain (7-point Likert scale), fatigue, head pain, emotional distress, abdominal pain, sense of relaxation, muscle pain, and sense of swelling, 3 times per day (morning, early afternoon, late afternoon)	Delayed	L
Page [107], 2010	14 (65.1)	No	**Pre:** PDQ-39, BDI-II, UPDRS	MPQ, in the medical appointment	Delayed	L
Palermo [33], 2004	30 IG (12.3 ± 2.4) 30 CG (12.3 ± 3.0)	Yes	**Pre:** CALI	Pain intensity (Faces pain scale [108]), pain symptoms (occurrence, location, duration, and emotional upset), CSI, and CALI, once a day	Delayed	H
Peters [109], 2000	80 (40.6 ± 6.7)	Yes	**Pre:** MPI, SF-36, BSI **Post:** CSQ (6 months follow up)	Pain intensity (7-point scale) and signal controlled diary (items: pain cognition, pain coping, sleep quality...), 4 times per day between 8 a.m. and 9:30 p.m.	Delayed	H
Roelofs [110], 2004	40 (46.4 ± 9.9)	Yes	**Pre:** TSK, QBPDS	Pain intensity (PVAQ), TSK, 8 times per day between 8 a.m. (weekend 9 a.m.) and 10 p.m.	Delayed	L
Schurman [35], 2010	10 IG 10 CG Overall (12.2 ± 2.8)	Yes	**Pre and Post:** BASC, PedsQL, completed by children and parents	Pain intensity (Faces pain scale Revised), once per day (bedtime) **CBT:** relaxation sessions, such as abdominal breathing, progressive muscle relaxation, imagery, and autogenic hand-warming. Multimedia content for home practice	Delayed	H
Sorbi [111], 2007	5	Yes		Pain intensity (VAS). 1st test run: 4–5 times per day. 2nd test run: 2–3 times per day **CBT:** migraine headache, medication use, attack precursors, self relaxation and other preventive behaviour	Instant	L
Stinson [112], 2008	Study 1 76 (13.4 ± 2.5) Study 2 36 (12.6 ± 2.4)	Yes	**Post:** PedsQL, PCQ	Pain intensity, pain unpleasantness, pain’s interference with aspects of quality of life and other symptoms (e.g., stiffness and fatigue) (VAS), 3 times per day (upon waking, after school, and before bed)	Instant	H
Stinson [113], 2012	24 children (5.9 ± 0.9) 77 youth (13.5 ± 3.1)	No		Pain intensity: faces pain scale (children), NRS (youth), in the medical appointment	Instant	H
Stone [31], 2003	40 IG (43.0 ± 9.0) 40 CG (48.0 ± 10.8)	Yes	**Pre:** MPQ-SF	BPI, PD-IIP, HAQ, 3 times per day (10 p.m., 4 a.m., 8 a.m.)	Delayed	H
Stone-Kelly [30,32], 2003	22 IG 3 prompts/day (49.0 ± 10.7) 22 IG 6 prompts/day (53.5 ± 10.4) 24 IG 12 prompts/day (50.3 ± 10.3) 23 CG (49.8 ± 12.5)	Yes	**Pre:** Questionnaire to assess anxiety, stress, pain, health, and quality of life **Pre/Treatment:** Questionnaire, once a week, to assess pain and mood, the momentary and the occurred over the last 7 days **Treatment:** Questionnaire once a week to assess interference of ED with participants' daily routines	Pain intensity (VAS), and other questions related to sensory, affective and physical aspects, 3, 6 or 12 times a day. Kelly’s study includes all the IGs	Delayed	H
Turner [47], 2005	61 IG (39.3 ± 11.1) 65 CG (35.4 ± 10.5)	Yes	**Pre:** GCPS	Pain intensity (NRS), pain-related activity interference, jaw use limitations, and several questions adapted from CSQ, SOPA, PCS, and DCI, 3 times per day (morning, afternoon, and evening) **CBT:** At each session activity goals were recommended (correct jaw posture, progressive relaxation practice, breathing exercises, physical exercise...)	Delayed	H
Wallasch [114], 2012	545 (43.1 ± 12.9)	Yes		MIDAS, GCPS, HADS, SF-12	Delayed	L
Weering [115], 2012	16 (40.7 ± 13.8).	Yes	**Pre:** RMDQ, SoC	Pain intensity (VAS), 3 times a day (noon, 4 p.m., 8 p.m.). Integration with Body Area Network (BAN)	Instant	L
Younger [116], 2009	10 (46.5 ± 10.3)	Yes	**Treatment:** FIQ every 2 weeks	Fibromyalgia severity, average pain intensity, highest pain, and other symptoms (fatigue, sadness, stress, sleep quality, ability to think and remember…), once a day (night)	NR	L
**Web-based systems**						
Berman [41], 2009	41 IG (64.3) 37 CG (67.5)		**Pre and Post:** BPI, PSEQ, CED-S, STAI, PAQ, HDM	Pain intensity (BPI), after logon and before logoff in the site **CBT:** abdominal breathing, relaxation, writing about experiences (positives or negatives), creative visual expression and positive thinking. Audio, visual and textual content related to pain		H
Buhrman [36], 2004	22 IG (43.5 ± 10.3) 29 CG (45.0 ± 10.7)		**Pre:** HADS	Pain intensity (VAS), 3 times per day (morning, noon and evening). PAIRS, MPI, CSQ and HADS once a week **CBT:** several modules (pain, stress, physical activities, problem solving...) and slideshows and sound files for download		H
Devineni [42], 2005	39 IG (43.6 ± 12.0) 47 CG (41.0 ± 11.8)			Frequency, duration, and severity of pain, once a day **Pre/Post/Follow up:** HSQ, CES-D, STAI, HDI **CBT:** muscle relaxation program, and stress coping therapy		H
Hicks [43], 2006	25 IG (12.1 ± 2.0) 22 CG (11.3 ± 2.2)		**Pre:** PedsQL **Post:** PedsQL (1-month and 3-month follow up)	Pain intensity (NRS), 4 times per day **CBT:** relaxation techniques, lifestyle (diet, exercise), information related to pain		H
Hunt [51], 2009	28 IG (39.0 ± 10.0) 26 CG (38.0 ± 12.0)			GSRS-IBS, IBS-QoL, ASI, GAD-Q and CPSQ, conducted at pre-and post-treatment and 3-month follow-up **CBT:** gastrointestinal symptoms and stress and on relaxation training, stress management, catastrophic thinking, exposure therapy and the social consequences of IBS		H
Kristjansdottir [52], 2011	6 (36.3)		**Pre and Post:** CPAQ, PCS	Pain intensity, interference of pain, planned and achieved activities, feelings, pain-related fear, avoidance, catastrophizing and acceptance, 3 times per day (morning, evening and a time randomly chosen between 11:30 a.m. and 2 p.m.) **CBT:** feedback SMS with praise, encouragement messages, and exercises		L
Ljótsson [45], 2010	42 IG (36.4 ± 10.1) 43 CG (32.8 ± 8.6)		**Treatment:** Gastrointestinal symptom diary	GSRS-IBS, IBS-QoL, VSI, MADRS-S and SDS conducted at pre-and post treatment. 3-month follow up: VSI, IBS-QoL and 2 weekly GSRS-IBS **CBT:** mindfulness exercises program, and lifestyle strategies (diet, exercise)		H
Lorig [39], 2008	422 IG (52.2 ± 10.9) 433 CG (52.5 ± 12.2)			Pre and post treatment, and 6/12 months follow up: pain intensity and fatigue (NRS), distress, activities limitations, disabilities and HAQ **CBT:** tailored exercises programmes and medication diaries		H
Palermo [37], 2009	26 IG (14.3 ± 2.1) 22 CG (15.3 ± 1.8)		**Pre and Post:** RCADS, ARCS	Pain intensity (NRS), CALI **CBT:** two separate websites, one for child access and one for parent access. The child access comprised eight treatment modules (education about chronic pain, recognizing stress and negative emotions, relaxation, distraction, cognitive skills, sleep hygiene and lifestyle, staying active, relapse prevention). Download of multimedia content.		H
Ruehlman [46], 2012	162 IG (19~78) 143 CG (19~78)			CES-D, DASS, PCP-S and PCP-EA at pre-treatment, 7-weeks and 14-weeks follow-up **CBT:** several content such as interactive activity, relaxation sessions		H
Strom [40], 2000	20 IG (41.5) 25 CG (39.2)		**Pre:** Pain intensity (VAS), duration, BDI, HDI, MLPC. **Treatment:** Number of times and the total time used for training relaxation. **Post:** Pain intensity (VAS)	**CBT:** several modules concerning relaxation		H
Williams [48], 2010	59 IG (50.2 ± 12.3) 59 CG (50.8 ± 10.6)		**Pre:** MINI, PD-IIP	SF-36, BPI, MFI, MOS-SS, CES-D, STPI and PGIC at pre and post-treatment **CBT:** multimedia content following topics: educational lectures, symptom management and adaptive life style		H

IG: Intervention Group; CG: Control Group; Q:Quality (H: Above average quality L: Below average quality); NR: Not Reported; ED: Electronic Diary; CBT: Cognitive-behavioural Therapy. ABC: Addiction Behaviours Checklist [117]; AMS: Analysys of Musculoskeletal Symptoms [118]; ARCS: Adult Responses to Children’s Symptoms Questionnaire [119]; ASEX: Arizona Sexual Experience [120]; ASI: Anxiety Sensitivity Index [121]; BASC: Behaviour Assessment System for Children [122]; BDI: Beck Depression Inventory [123]; BDI-II: BDI revised; BFI: Brief Fatigue Inventory [124]; BPI: Brief Pain Inventory [125]; BSI: Brief Symptom Inventory [126]; CALI: Child Activity Limitations Interview [127]; CALQ: Child Activity Limitations Questionnaire [128]; CES-D: Center for Epidemiological Studies Depression Scale [129]; CPAQ: Chronic Pain Acceptance Questionnaire [130]; CPEQ: Comprehensive Pain Evaluation Questionnaire [131]; CPSQ: Consequences of Physical Sensations Questionnaire [132]; COMM: Current Medication Misuse Measure [133]; CSI: Children’s Somatisation Inventory [134]; CSQ: Coping Strategies Questionnaire [135]; CSFBD: Cognitive Scale for Functional Bowel Disorders [136]; DASS: Depression Anxiety Stress Scale [137]; DCI: Daily Coping Inventory [138]; EQ5D: Euro-QoL 5 [139]; FIQ: Fibromyalgia Impact Questionnaire [140]; GAD-Q: Generalized Anxiety Disorder Questionnaire [141]; GCPS: Graded Chronic Pain Scale [142]; GSRS-IBS: Gastrointestinal Symptom Rating Scale—Irritable Bowel Syndrome [143]; IBS-QoL: Irritable Bowel Syndrome Quality of Life [144]; IPAQ: International Physical Activity Questionnaire [145]; HADS: Hospital Anxiety and Depression Scale [146]; HAQ: Health Assessment Questionnaire [147]; HDI: Headache Disability Inventory [148]; HDM: Healthy Days Measures [149]; HSCL-25: Hopkins Symptom Check List [150]; HSQ: Headache Symptom Questionnaire [151]; MADRS-S: Montgomery Åsberg Depression Rating Scale-Self report [152]; MASQ: Multiple Ability Self-Report Questionnaire [153]; MDHAQ: Multidimensional Health Assessment Questionnaire [154]; MFI: Multidimensional Fatigue Inventory [155]; MHAQ: Modified Health Assessment Questionnaire [156]; MIDAS: MIgraine Disability Assessment Score [157]; MINI: Mini-International Neuropsychiatric Interview [158]; MLPC: Multidimensional Locus of Pain Control [159]; MOS-SS: Medical Outcomes Study Sleep Scale [160]; MPI: Multidimensional Pain Inventory [161]; MPQ: McGill Pain Questionnaire [162]; MPQ-SF: MPQ-Short Format; NRS: Numeric Rating Scale [163]; ODI: Oswestry Disability Index [164]; PAIRS: Pain Impairment Rating Scale [165]; PANAS: Positive and Negative Affect Schedule [166]; PANAS-C: PANAS for Children; PAQ: Pain Awareness Questionnaire [41]; PCP-EA: Profile of Chronic Pain Extended Assessment [167]; PCP-S: Profile of Chronic Pain: Screen [168]; PCQ: Pain Coping Questionnaire [169]; PCS: Pain Catastrophizing Scale [170]; PD-IIP: Personality Disorders Scale of the Inventory of Interpersonal Problems [171]; PD-Q: painDETECT questionnaire [172]; PDI: Pain Disability Index [173]; PDQ-39: Parkinson’s Disease Questionnaire-39 [174]; PDUQ: Prescription Drug Use Questionnaire [175]; PedsQL: Pediatric Quality of Life Inventory [176]; PGIC: Patient Global Impression of Change [177]; PHQ: Patient Health Questionnaire [178]; PSEQ: Pain Self-efficacy Questionnaire [179]; PVAQ: Pain Vigilance and Awareness Questionnaire [180]; QBPDS: Quebec Back Pain Disability Scale [181]; RADAI: Rheumatoid Arthritis Disease Activity Index [182]; RCADS: Revised Child Anxiety and Depression Scale [183]; RMDQ: Roland Morris Disability Questionnaire [184]; SCL-90: Symptom Checklist-90 [185]; SDS: Sheehan Disability Scale [186]; SF-36: MOS 36-ltem short-form [187] (SF-12 are a short version of SF-36); SOAPP-R: Screener and Opioid Assessment for Pain Patients-Revised [188]; SoC: Stage of Change [189]; SOPA: Survey of Pain Attitudes [190] ; STAI: State-Trait Anxiety Inventory [191]; STPI: State-Trait Personality Inventory [192]; TSK: Tampa Scale for Kinesiophobia [193]; UPDRS: Unified Parkinson’s Disease Rating Scale [194]; VAS: Visual Analogue Scale [195]; VSI: Visceral Sensitivity Index [196].

## Appendix A2

The quality assessment tool which includes: (1) Formulation of the research question; (2) Specification of inclusion/exclusion criteria; (3) Sample description; (4) Design; (5) Technical description; (6) Description of study procedure; (7) Statistical analyses; (8) Conclusions supported by data; (9) Limitations of study analyzed explicitly; (10) Research questions are answered.

## Appendix A3

**Table A2:** Risk of bias assessment.

Study/Year	Sequence Generation	Allocation Concealment	Blinding of Participants, Personnel and Outcome Assessors	Incomplete Outcome Data	Free of Selective Outcome Reporting	Free of Other Sources of Bias
Berman [41], 2009	Yes	No	No	Yes	Yes	No
Buhrman [36], 2004	Yes	Yes	No	Yes	Yes	Yes
Devineni [42], 2005	No	Yes	No	Yes	Yes	Yes
Hicks [43], 2006	Yes	Yes	No	No	Yes	Yes
Hunt [51], 2009	Yes	Yes	No	No	Yes	No
Litt [44], 2009	Yes	Yes	No	Unclear	Yes	Yes
Ljótsson [45], 2010	Yes	Yes	No	Unclear	Yes	Yes
Lorig [39], 2008	Yes	Yes	No	No	Yes	Yes
Marceau [105], 2010	Yes	Yes	No	No	Yes	Yes
Oerlemans [38], 2011	Yes	Yes	No	Unclear	Yes	No
Palermo [37], 2009	Yes	Yes	No	Yes	Yes	Yes
Ruehlman [46], 2012	Yes	Yes	No	Yes	Yes	No
Schurman [35], 2010	Yes	Yes	No	Yes	Yes	Yes
Strom [40], 2000	Yes	Yes	No	Yes	Yes	No
Turner [47], 2005	No	Yes	No	Yes	Yes	Yes
Williams [48], 2010	Yes	Yes	No	Unclear	Yes	Yes
